# Association of Gastric Microbiota with Gastric Cancer in Patients Infected with *Helicobacter pylori*

**DOI:** 10.3390/pathogens15101051

**Published:** 2026-10-02

**Authors:** Bekir Kocazeybek, Kadir Bal, Seher Akkus, Mehmet Demirci, Enes Ali Kurt, Nesrin Gareayaghi, Suleyman Demiryas, Dogukan Ozbey, Nuray Kepil, Tevhide Ziver Sarp, Harika Oyku Dinc, Oguz Kagan Bakkaloglu, Huseyin Cakan, Ferhat Osman Dasdemir, Ruveyda Akcin, Suat Saribas, Onder Yuksel Eryigit, Hrisi Bahar Tokman

**Affiliations:** 1 Department of Medical Microbiology, Cerrahpasa Medical Faculty, Istanbul University-Cerrahpasa, Istanbul 34320, Turkey; dr.seher@hotmail.com (S.A.); dogukanozbey@gmail.com (D.O.); suatsaribas@gmail.com (S.S.); hrisibahar@gmail.com (H.B.T.); 2 Department of Gastroenterology, Cerrahpasa Medical Faculty, Istanbul University-Cerrahpasa, Istanbul 34320, Turkey; kadirbal@iuc.edu.tr (K.B.); drenesalikurt@hotmail.com (E.A.K.); oguzkagan.bakkaloglu@iuc.edu.tr (O.K.B.); 3 Department of Medical Microbiology, Kırklareli University Medical Faculty, Kırklareli 39000, Turkey; mdemirci1979@gmail.com; 4 Center for Blood, Istanbul Sisli Hamidiye Etfal Training and Research Hospital, University of Health Sciences, Istanbul 34668, Turkey; nesrin.gareyaghi@sbu.edu.tr; 5 Department of General Surgery, Cerrahpasa Medical Faculty, Istanbul University-Cerrahpasa, Istanbul 34320, Turkey; drdemiryas@yahoo.com; 6 Medical Microbiology Department, Faculty of Medicine, Istanbul Okan University, Istanbul 34959, Turkey; 7 Department of Pathology, Cerrahpasa Medical Faculty, Istanbul University-Cerrahpasa, Istanbul 34320, Turkey; nuray.kepil@iuc.edu.tr; 8 Department of Nutrition and Dietetic Famagusta, Faculty of Health Sciences, Eastern Mediterranean University, Mersin 99628, Turkey; tevhide_ziver@hotmail.com; 9 Department of Microbiology, Medical Faculty, Uskudar University, Istanbul 34662, Turkey; oykudinc@gmail.com; 10 Biology Division, Basic and Industrial Microbiology, Faculty of Science, Çanakkale Onsekiz Mart University, Canakkale 17020, Turkey; huseyin.cakan@comu.edu.tr; 11 Department of Environmental Protection Technologies, Vocational School, Istanbul Arel University, Istanbul 34537, Turkey; ferhatosmandasdemir@arel.edu.tr; 12 Department of Microbiology, Medical Faculty, Istanbul Health and Technology University (İSTÜN), Istanbul 34275, Turkey; ruveydaakcin@gmail.com; 13 Department of Anesthesiology and Reanimation, Medical Faculty, Istanbul Health and Technology University (İSTÜN), Istanbul 34275, Turkey; onder.eryigit@istun.edu.tr

**Keywords:** *Helicobacter pylori*, gastric microbiota, *Enterobacteriaceae*, gastric cancer, 16S rRNA sequencing

## Abstract

**Background**: The association between *Helicobacter pylori* and the resident gastric microbiota plays a critical role in gastric carcinogenesis; however, the specific microbial alterations accompanying the transition from chronic inflammation to malignancy remain to be fully elucidated. This study aimed to characterize the distinct bacterial signatures associated with gastric cancer (GC) and non-ulcer dyspepsia (NUD) within a Turkish cohort, evaluating these structural shifts in the context of the “hit-and-run” hypothesis. **Methods:** Genomic DNA was isolated from 158 gastric biopsy specimens obtained from 96 individuals stratified into four homogeneous groups based on clinical *H. pylori* status and histopathological presentation: *H. pylori*-positive GC (Hp+GC, *n* = 37), *H. pylori*-negative GC (Hp-GC, *n* = 25), *H. pylori*-positive NUD (Hp+NUD, *n* = 17), and *H. pylori*-negative NUD (Hp-NUD, *n* = 17). Next-generation amplicon sequencing targeting the V3–V4 hypervariable regions of the 16S rRNA gene was executed, followed by strict quality filtering via the DADA2 pipeline and taxonomic classification against the SILVA database. **Results**: Alpha diversity analysis demonstrated a significant reduction in microbial richness and evenness within the Hp+GC group compared with all other cohorts (FDR-adjusted q < 0.001). Beta diversity metrics revealed distinct ecological trajectories, with the overall microbial community structure of the Hp+GC group diverging sharply from non-malignant controls. Taxonomic profiling unveiled a profound dysbiotic shift in the Hp+GC microenvironment, characterized by a massive depletion of the Firmicutes phylum and a concomitant overrepresentation of *Proteobacteria* (66.56%). While the *Helicobacter* genus predominated in the non-malignant Hp+NUD group, its relative abundance declined to negligible levels (<0.01%) in biopsy samples of gastric cancer patients, despite clinical positivity. This malignant niche was instead robustly invaded by potentially pathogenic bacteria belonging to the *Enterobacteriaceae* family, showing highly significant enrichment of *Morganella* (11.14% mean relative abundance; FDR-adjusted q < 0.001), *Providencia* (3.43%; FDR-adjusted q < 0.001), and *Klebsiella* (2.03%; FDR-adjusted q < 0.001). **Conclusions:** These findings support the “hit-and-run” model, indicating that *H. pylori* acts as an initiator of carcinogenesis but is progressively outcompeted by an aggressive, nitrate-reducing, and pro-inflammatory *Enterobacteriaceae*-dominated dysbiotic bacterial community as mucosal atrophy and hypochlorhydria neutralize the typical acidic gastric barrier.

## 1. Introduction

Gastric cancer (GC) remains one of the leading causes of cancer-related morbidity and mortality globally, and its etiology involves a complex interplay between genetic susceptibility, environmental factors, and microbial agents [1,2]. Among these, *Helicobacter pylori* infection has been identified as the most prominent risk factor in gastric carcinogenesis and is classified as a Group I carcinogen by the World Health Organization [3]. In the histopathological cascade, also known as the Correa cascade, *H. pylori*-induced chronic inflammation may progress sequentially to atrophic gastritis, intestinal metaplasia, dysplasia, and ultimately adenocarcinoma [4]. However, only a small proportion (1–3%) of *H. pylori*-infected individuals develop GC; hence, together with the geographical discrepancies between infection prevalence and cancer incidence, this indicates that additional components of the resident gastric microbiota may play a crucial role in this process [5,6].

Historically, the stomach was considered a sterile or microbiologically insignificant organ due to its low pH and strong peristaltic activity [7]. However, advances in culture-independent next-generation sequencing technologies, particularly 16S rRNA gene sequencing, have demonstrated that the stomach harbors a distinct and dynamic microbial ecosystem [8]. The acidic environment in a healthy stomach acts as an effective physiological barrier against pathogenic colonization, and a balanced distribution of bacterial phyla, including *Firmicutes*, *Bacteroidetes*, *Actinobacteria*, *and Proteobacteria*, is maintained [9]. During gastric carcinogenesis, glandular atrophy and loss of parietal cells reduce acid secretion (hypochlorhydria), thereby weakening this barrier and enabling colonization by bacteria that are not normally present in the gastric niche [10]. This shift promotes a dysbiotic state within the gastric microbiota that may enable tumor development.

The increasingly recognized “hit-and-run” hypothesis in gastric microbiota research proposes that *H. pylori* acts as the primary initiator of carcinogenesis but is gradually replaced by other opportunistic pathogens as the tumor microenvironment evolves [11]. *H. pylori* colonization declines, whereas bacteria capable of nitrate reduction and possessing genotoxic potential—including *Lactobacillus*, *Streptococcus*, *Veillonella*, and members of the *Enterobacteriaceae* family—become more prevalent in advanced GC and severe atrophic gastritis [12,13]. This dysbiotic microbial milieu may accelerate carcinogenesis by worsening inflammatory responses, inducing severe oxidative stress, and promoting DNA damage through the production of genotoxic metabolites and N-nitroso compounds [14]. Therefore, focusing not only on the presence of *H. pylori* but also on the overall compositional alterations in the bacterial community and the emergence of dominant opportunistic taxa is crucial for understanding GC pathogenesis.

Geographic location, dietary habits, and host genetic background strongly affect the composition of the gastric microbiota [15]. Several studies have compared microbiota profiles in *H. pylori*-positive individuals and across different clinical conditions, including GC and non-ulcer dyspepsia (NUD); however, comprehensive data from the Turkish population remain limited. The present study aimed to characterize alterations in the gastric bacterial community according to *H. pylori* status and disease stage (NUD vs. GC) in a Turkish cohort using 16S rRNA amplicon sequencing. This study seeks to reveal potential bacterial biomarkers that may contribute to the early diagnosis or prognosis of GC by identifying disease-associated bacterial taxa and dysbiotic patterns.

## 2. Materials and Methods

### 2.1. Study Design and Patients

This case–control study was conducted utilizing gastric biopsy tissue samples collected from 96 distinct adult patients. To provide a comprehensive representation of the gastric niche while ensuring absolute statistical independence for downstream inferential models, separate biopsy specimens were collected from both the antrum and the corpus of each individual, yielding a total of 158 distinct tissue samples. Prior to downstream sequencing and library preparation, the genomic DNA extracts derived from the antrum and corpus of each corresponding patient were pooled into a single, patient-specific composite sample. Consequently, each patient is treated as a single, independent unit of observation (effective sample size, *n* = 96), successfully mitigating the risk of within-patient correlation artifacts or *p*-value inflation.

Clinical *H. pylori* status was established via histopathological evaluation using Giemsa staining and the Rapid Urease Test (RUT). A patient was defined as *H. pylori*-positive if at least one of these diagnostic evaluations yielded a positive result, thereby confirming active infection rather than historical exposure. In our histopathological evaluation using the updated Sydney classification, *H. pylori* colonization was assessed qualitatively and semi-quantitatively. In the Hp+NUD cohort, the majority of biopsies demonstrated moderate-to-marked bacterial density clinging to intact surface mucus and foveolar epithelium. Conversely, in the Hp+GC group, while bacterial presence was confirmed microscopically (primarily patchy, low-density colonization in remaining non-neoplastic atrophic mucosa) or via RUT, the bacterial load was markedly sparse over neoplastic, metaplastic, or ulcerated surfaces. The biopsies used for microbiota sequencing were obtained from the antrum and corpus [these were tumor tissue, in cancer patients]. Based on clinical and histopathological classifications (including the Sydney and World Health Organization systems), patients were stratified into four distinct, homogeneous groups: *H. pylori*-positive gastric cancer (Hp+GC, *n* = 37), *H. pylori*-negative gastric cancer (Hp-GC, *n* = 25), *H. pylori*-positive non-ulcer dyspepsia (Hp+NUD, *n* = 17), and *H. pylori*-negative non-ulcer dyspepsia (Hp-NUD, *n* = 17). NUD cohorts were strictly matched with the study groups (GC cohorts) regarding age and gender distribution (*p* > 0.05). We used NUD (non-ulcer dyspepsia) to describe functional dyspepsia. The symptoms of patients with NUD included bothersome postprandial fullness, early satiation-epigastric pain, and burning with no evidence of structural disease (including at upper endoscopy).

Exclusion criteria comprised patients younger than 18 years, individuals with a history of prior gastric surgery or *H. pylori* eradication therapy, and those who had received antibiotics, antisecretory agents (e.g., proton pump inhibitors), bismuth salts, or sucralfate within one month prior to endoscopy. Biopsy samples were collected in *Brucella* broth during upper gastrointestinal endoscopy and immediately stored at −80 °C. All participants provided written informed consent, and the study design was formally approved by the Istanbul University Cerrahpasa Clinical Trials Ethics Committee (Approval No: A-57, approval date: 2 April 2019).

All biopsy specimens designated for molecular sequencing, histopathological examination (Giemsa staining according to the updated Sydney system), and rapid urease testing were collected during the exact same endoscopic procedure.

### 2.2. DNA Isolation and Contamination Control

Total genomic DNA was isolated using DNeasy Blood and Tissue Kits (Qiagen GmbH, Hilden, Germany) in strict accordance with the manufacturer’s operational instructions. DNA quantification was performed using a Qubit^®^ 2.0 fluorometer (Thermo Scientific, Waltham, MA, USA), and purified extracts were stored at −20 °C. Given that gastric biopsy specimens represent relatively low-biomass environments susceptible to exogenous DNA contamination, rigorous negative controls may reduce the likelihood of contamination-related artifacts. Extraction blanks (substituting sterile nuclease-free water for tissue) and PCR no-template controls (NTCs) were processed concurrently alongside every batch of clinical samples. These negative controls exhibited no discernible gel electrophoresis bands or significant fluorometric amplification. Any low-frequency background sequences identified within these control blanks were bioinformatically filtered out during the DADA2 processing pipeline to safeguard the authenticity of the biological signals.

### 2.3. Library Preparation and Sequencing

Bacterial 16S rRNA gene amplicon libraries were constructed targeting the V3–V4 hypervariable regions. We utilized the locus-specific forward primers (5′-TCG TCG GCA GCG TCA GAT GTG TAT AAG AGA CAG CCT ACG GGN GGC WGC AG-3′) and reverse primers (5′-GTC TCG TGG GCT CGG AGA TGT GTA TAA GAG ACA GGA CTA CHV GGG TAT CTA ATC C-3′), which represent a highly efficient universal eubacterial primer set as validated by Klindworth et al. [9]. Library preparation followed a standardized two-step polymerase chain reaction (PCR) protocol executed on a T100 Thermal Cycler (Bio-Rad Laboratories, Hercules, CA, USA). The first-stage PCR utilized the KAPA HiFi HotStart ReadyMix Kit (Roche Diagnostics GmbH, Mannheim, Germany) under the following thermal parameters: initial denaturation at 95 °C for 3 min; 25 cycles of denaturation at 95 °C for 30 s, annealing at 55 °C for 30 s, and extension at 72 °C for 30 s; followed by a final extension at 72 °C for 5 min. 

The second-stage PCR was performed to incorporate dual-indexing adapters using the Nextera XT Index Primer 1 and Primer 2 sets (Illumina, San Diego, CA, USA). The thermal profile consisted of 95 °C for 3 min; 8 cycles of 95 °C for 30 s, 55 °C for 30 s, and 72 °C for 30 s; with a final extension at 72 °C for 5 min. Amplicon purification after each PCR stage was conducted using the Agencourt AMPure XP magnetic bead kit (Beckman Coulter Life Sciences, Indianapolis, IN, USA) following the manufacturer’s protocol. Library fragment selection and relative density were verified via 2% agarose gel electrophoresis. Following library quantification and equimolar normalization using the Qubit fluorometer, bidirectional sequencing (2 × 150 bp) was executed on the Illumina iSeq 100 next-generation sequencing platform (Illumina, San Diego, CA, USA) utilizing the iSeq 100 i1 Reagent Kit (Illumina, San Diego, CA).

### 2.4. Bioinformatic and Statistical Analysis

Demographic and clinical descriptive statistics were analyzed using Statistical Package for the IBM SPSS Statistics for Windows, Version 20.0 (IBM Corp., Armonk, NY, USA). Categorical variables were evaluated via the Chi-square test, while continuous variables were modeled using either Student’s *t*-test or the Mann–Whitney U test depending on normality distributions. 

Raw FASTQ sequencing reads were bioinformatically processed using the Quantitative Insights Into Microbial Ecology 2 (QIIME2, version 2022.4) platform. Quality control, sequence denoising, read merging, and chimera identification were executed utilizing the DADA2 plugin. Forward and reverse reads were truncated at positions where the median Phred quality score dropped below 20 to ensure high-quality base calls. The maximum expected error threshold was set to a standard value of 2.0 (maxEE = 2), and chimeras were discarded using the “consensus” method. Read merging resulted in a final feature table encompassing 3245 unique Amplicon Sequence Variants (ASVs). Taxonomic assignment was performed via a pre-trained Naive Bayes machine-learning classifier trained specifically on the V3–V4 regions of the 16S rRNA gene derived from the SILVA reference database (v138) clustered at 99% similarity, applying a standard confidence threshold of 0.7. Samples failing to meet minimum sequencing depth thresholds were excluded. To eliminate library size bias prior to diversity analyses, the feature table was rarefied to an even sampling depth [16].

Downstream statistical modeling and data visualizations were performed in a Python (v3.8) environment utilizing the pandas, scipy, scikit-bio, and seaborn libraries. Alpha diversity within samples was evaluated using the Shannon, Simpson, Chao1 (richness), and Pielou (evenness) indices, with cross-cohort differences determined via the Kruskal–Wallis H test followed by post hoc pairwise comparisons. Beta diversity across cohorts was assessed via Bray–Curtis and Jaccard distance matrices calculated from the rarefied ASV tables. Structural community clustering was visualized using Principal Coordinates Analysis (PCoA). The statistical significance of the ecological separation among the four clinical groups was rigorously verified using Permutational Multivariate Analysis of Variance (PERMANOVA) with 999 permutations. 

Prior to differential abundance testing, raw ASV counts were normalized via Total Sum Scaling (TSS) to generate relative abundance values. To minimize zero-inflation and sparsity artifacts, rare taxa with an overall relative abundance below 0.1% or those present in fewer than 10% of the total samples were removed. Differences in taxonomic relative abundances across the four cohorts were evaluated using the Mann–Whitney U test. To maintain strict control over type I error inflation due to multiple parallel hypotheses, all resulting taxonomic, alpha, and beta-diversity *p*-values were rigorously adjusted using the Benjamini–Hochberg False Discovery Rate (FDR) procedure. FDR corrections were executed separately for each discrete test family (i.e., phylum-level tests, genus-level tests, and pairwise diversity contrasts were corrected as independent families). All reported values in the text, tables, and figures represent these FDR-adjusted q-values, with a significance threshold defined at q < 0.05. For heatmap visualizations, relative abundances were log10-transformed to enable clear resolution of both high- and low-abundance dominant bacterial genera.

Overall Testing Hierarchy (Omnibus test followed by post hoc pairwise testing):

Our statistical strategy strictly followed a two-stage hierarchical model:

- Stage 1 (Global omnibus test): For both alpha diversity indices and taxonomic relative abundances (at phylum and genus levels), an initial non-parametric Kruskal–Wallis H test across all four clinical cohorts (Hp+GC, Hp−GC, Hp+NUD, and Hp−NUD) was conducted to determine whether overall inter-group variance was statistically significant.
- Stage 2 (Post hoc pairwise testing): Only taxa or diversity indices demonstrating global significance in the omnibus test were subjected to post hoc pairwise contrasts using the two-tailed Mann–Whitney U test. Primary biological comparisons focused on: (i) Hp+GC vs. Hp−GC (evaluating *H. pylori* impact in malignancy), (ii) Hp+GC vs. Hp+NUD (evaluating malignant transition under active infection), and (iii) Hp+GC vs. Hp−NUD (comparing cancer with baseline non-malignant controls).

FDR Correction and Test Family Definition:

- False discovery rate (FDR) control was executed via the Benjamini–Hochberg procedure. Crucially, corrections were structured independently across discrete biological test families:
- All genus-level comparisons constituted one comprehensive test family where all *p*-values were adjusted jointly.
- Phylum-level tests and pairwise alpha/beta diversity comparisons formed separate test families.
- All values reported in the text, figures, and tables represent these FDR-adjusted q-values (significance set at q < 0.05).

Compositionality Considerations and Justification of Approach:

We explicitly acknowledge that 16S amplicon relative-abundance data are constrained by a constant sum (compositional nature), which can theoretically induce spurious correlations. While specialized Compositional Data Analysis (CoDA) tools (e.g., ANCOM-BC or ALDEx2) exist, we adopted Total Sum Scaling (TSS) combined with non-parametric Mann–Whitney U/Kruskal–Wallis testing after stringent low-abundance filtering (<0.1\% abundance and <10\% sample prevalence). This approach is robust against zero-inflation and non-normality and directly reflects the large effect sizes observed in dominant taxa (e.g., *Morganella* and *Proteobacteria* expansions), avoiding model-dependent parametric assumptions. We explicitly acknowledge microbiome compositionality and reaffirm this justification in both Section 2 and Section 4.

## 3. Results

### 3.1. Sequencing Metrics and Cohort Characteristics

**Figure 1:**
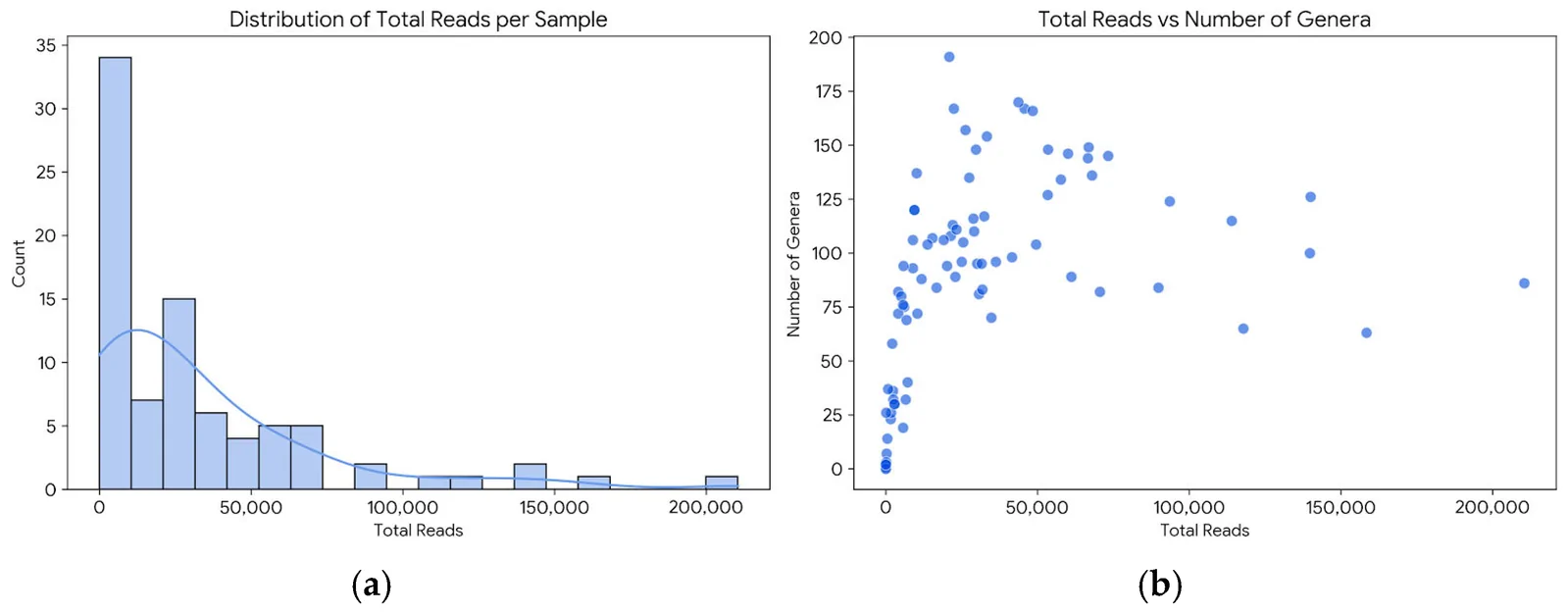
Distribution of sequencing depth across samples and the association between read counts and detected taxonomic richness. (**a**) Histogram demonstrating the frequency distribution of total raw read counts (total reads) obtained per sample after DADA2 processing. (**b**) Scatter plot illustrating the correlation between total high-quality read counts and the number of detected bacterial genera (genus) in the corresponding composite samples.

Following initial next-generation sequencing, a total of 13,248,600 raw reads were generated across all clinical samples. After undergoing rigorous quality control filtering—including the truncation of sequences with a Phred quality score below 20, sequence denoising, read merging, and consensus-based chimera removal via the DADA2 algorithm—a total of 9,125,448 high-quality reads were successfully retained. The final downstream analysis included robust, filtered sequencing data from 158 gastric biopsy specimens collected from 96 individual participants, which were bioinformatically pooled patient-specifically to maintain clinical independence. Among the sequenced samples, the per-sample read depth ranged from a minimum of 18,240 to a maximum of 1,138,855 reads, with an overall mean depth of 57,756 and a median depth of 34,500 reads per sample (Figure 1).

Patients were stratified into four distinct clinical groups based on their active *H. pylori* status and histopathological presentation: *H. pylori*-positive gastric cancer (Hp+GC, *n* = 37), *H. pylori*-negative gastric cancer (Hp-GC, *n* = 25), *H. pylori*-positive NUDa (Hp+NUD, *n* = 17), and *H. pylori*-negative NUD (Hp-NUD, *n* = 17). Table 1 presents the primary demographic and clinical baseline characteristics of the study population. Initial amplicon-based taxonomic profiling identified a total of 42 phyla, 62 classes, 99 orders, 256 families, and 881 distinct genera across the patient cohorts prior to downstream quality filtering of non-bacterial sequences.

### 3.2. Microbial Diversity and Ecological Dissimilarity

**Figure 2:**
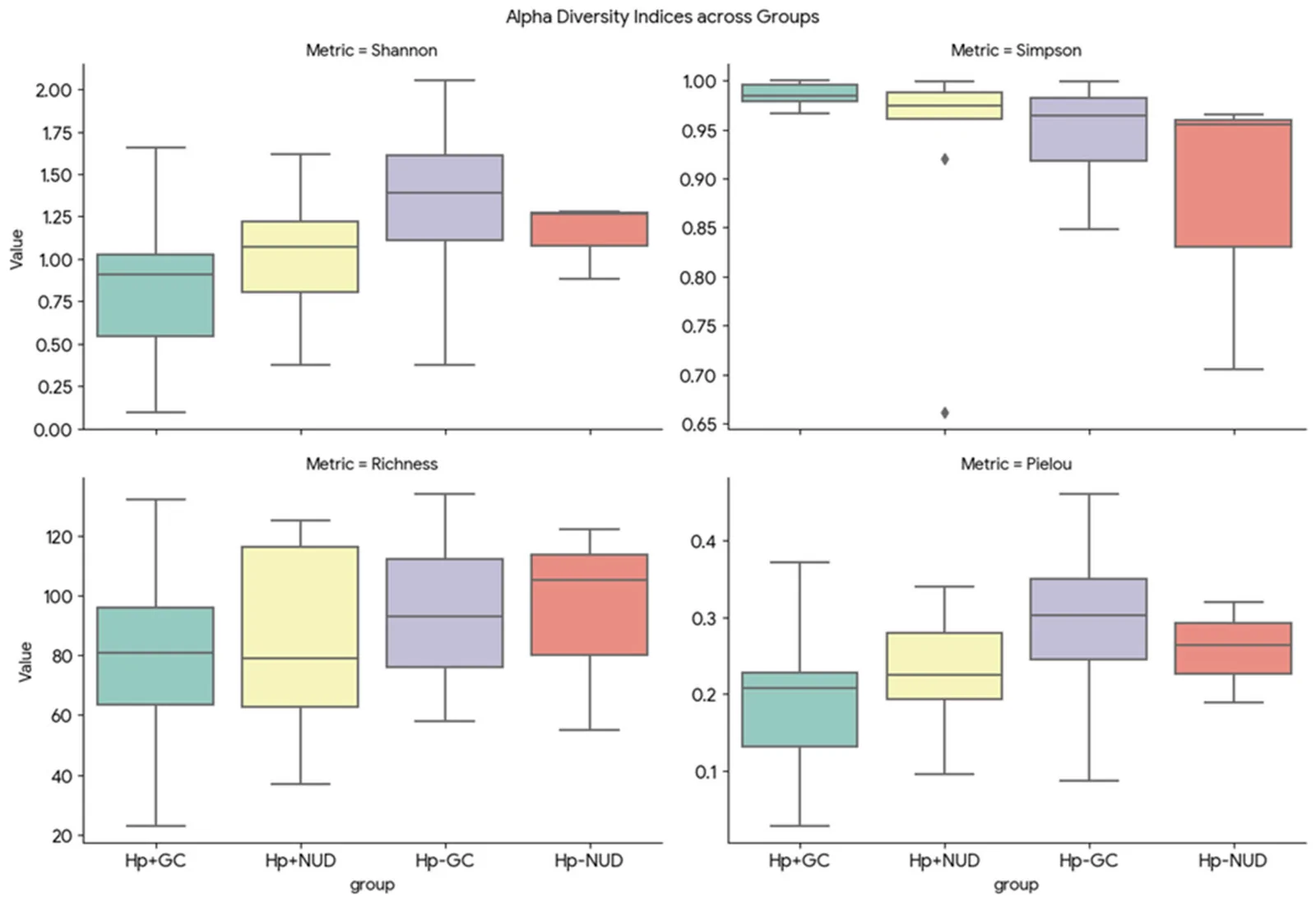
Comparison of the alpha diversity indices of the gastric mucosal microbiota among the study groups. The panel presents alpha diversity metrics across the cohorts, evaluating within-sample microbial diversity, richness, and evenness (Shannon index, Simpson index, Observed Richness, and Pielou’s evenness). Box plots denote the median (horizontal line), interquartile range (box boundaries), and minimum–maximum values (whiskers). All reported *p*-values represent Benjamini–Hochberg FDR-adjusted q-values (q < 0.001). Abbreviations: Hp+GC, *H. pylori*-positive gastric cancer; Hp+NUD, *H. pylori*-positive non-ulcer dyspepsia; Hp−GC, *H. pylori*-negative gastric cancer; Hp−NUD, *H. pylori*-negative non-ulcer dyspepsia.

Microbial community diversity analysis within individual samples (alpha diversity) revealed highly significant differences among the four study cohorts. According to the Shannon and Pielou’s evenness diversity indices, the malignant Hp+GC group demonstrated the lowest alpha diversity compared with all other groups, and this ecological collapse was highly significant (Kruskal–Wallis, FDR-adjusted q < 0.001) (Figure 2). Conversely, the highest overall microbial richness and diversity were observed in the non-malignant, *H. pylori*-negative control profile (Hp-NUD group).

**Figure 3:**
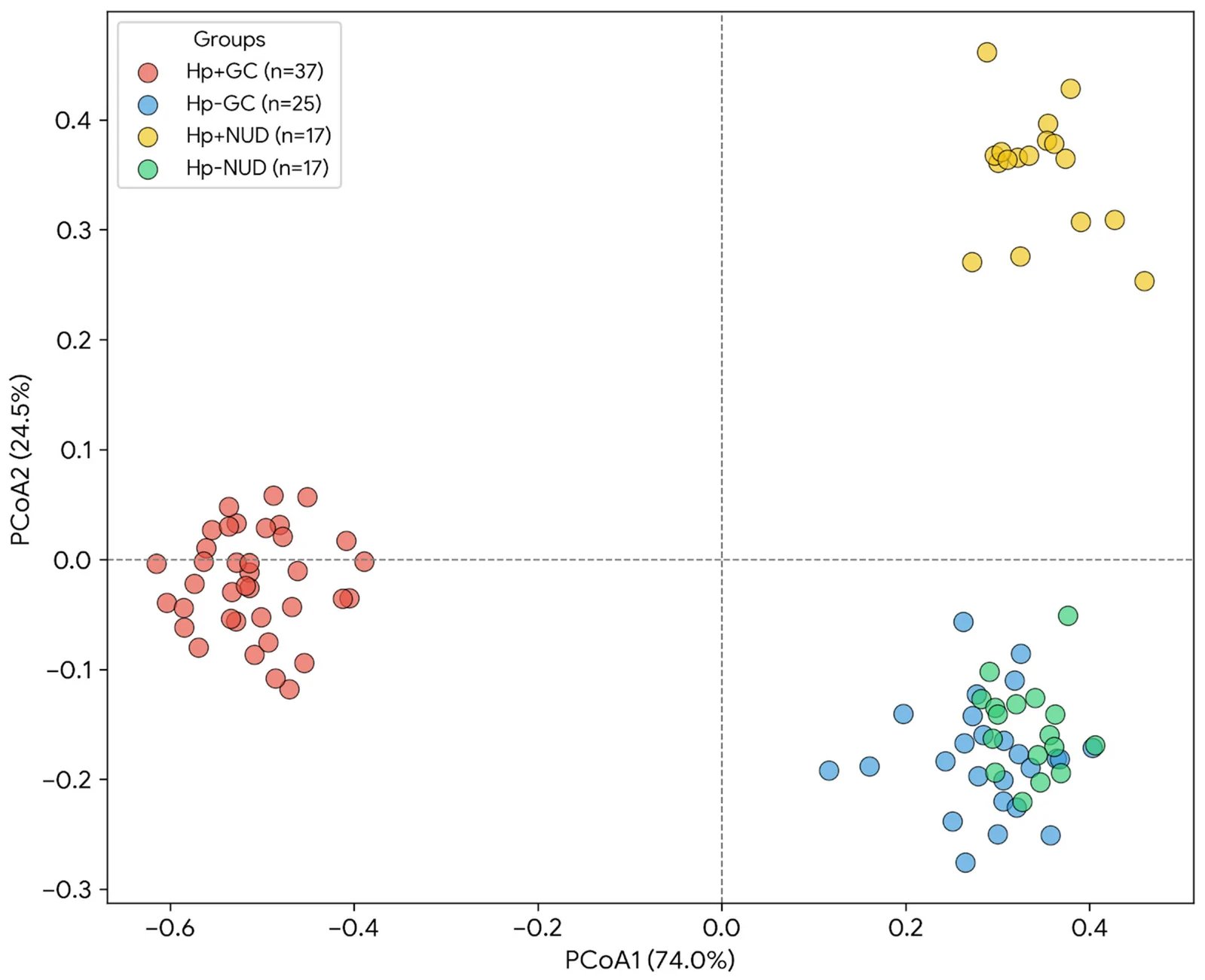
Sample-level Principal Coordinates Analysis (PCoA) based on the Bray–Curtis distance matrix. Each individual data point represents a patient-specific composite gastric bacterial profile. The malignant Hp+GC cohort (red circle; *n* = 37) demonstrates a distinct ecological separation and structural trajectory away from the Hp-GC cohort (blue; *n* = 25), Hp+NUD cohort (yellow; *n* = 17), and Hp-NUD cohort (green; *n* = 17), illustrating a unique pathological niche (global PERMANOVA *p* < 0.001).

Evaluations of the overall microbial community structure between samples (beta diversity) demonstrated macroscopic ecological divergence between the malignant and non-malignant states. Principal Coordinates Analysis (PCoA) based on Bray–Curtis and Jaccard distance metrics, rigorously verified by Permutational Multivariate Analysis of Variance, confirmed that the clinical groups formed highly distinct clusters in multivariate space (global PERMANOVA, pseudo-F = 45.43, *p* < 0.001) (Figure 3). Pairwise PERMANOVA tests confirmed that the overarching ecological trajectory of the Hp+GC group diverged remarkably from the other three cohorts (pairwise adjusted q < 0.001), representing a unique and highly altered niche driven by late-stage tumor progression.

### 3.3. Taxonomic Profiling at the Phylum Level

**Figure 4:**
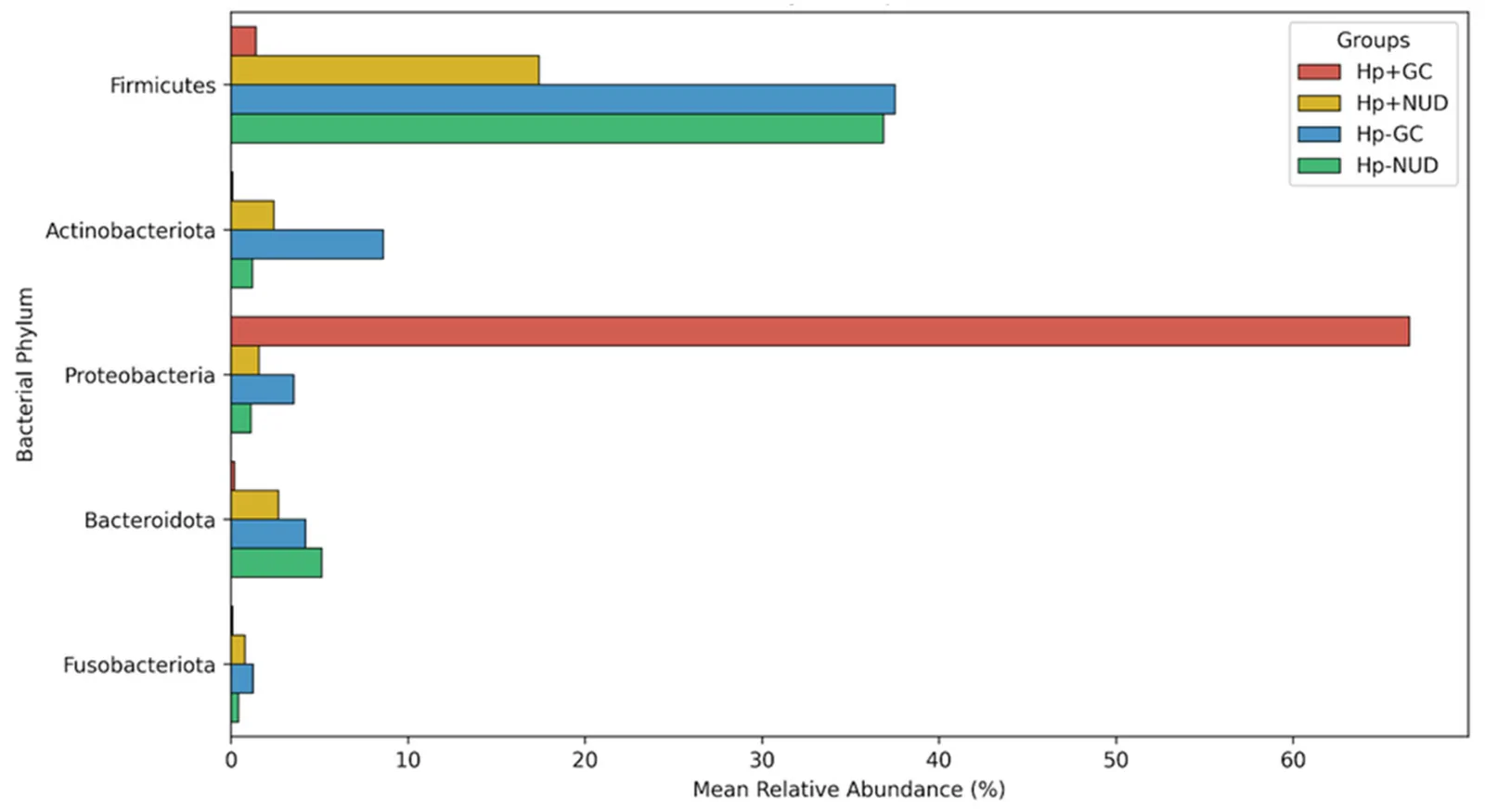
Taxonomic composition and relative abundance of the gastric microbiota at the phylum level across the study groups. Horizontal bars demonstrate the mean relative abundance (%) of genuine, filtered bacterial phyla. Non-bacterial lineages and off-target eukaryotic sequences have been bioinformatically removed to safeguard biological accuracy. All reported cross-cohort differences are validated using Benjamini–Hochberg FDR-adjusted q-values (q < 0.001).

Across all study groups, the dominant bacterial phyla within the human gastric mucosa included *Proteobacteria*, *Firmicutes*, *Bacteroidota*, *Actinobacteriota*, and *Fusobacteriota* (Figure 4). The relative abundance and distribution of these phyla differed dramatically among the groups as a consequence of microenvironmental shifts (FDR-adjusted q < 0.001).

The gastric microbiota of the cancerous Hp+GC group was severely dominated by the *Proteobacteria* phylum, which accounted for a striking mean relative abundance of 66.56% of the total community. Concurrently, the proportion of the commensal *Firmicutes* phylum underwent a dramatic collapse, dropping to a mere 1.40% in the Hp+GC cohort. In sharp contrast, Hp-NUD group maintained a balanced, healthy-like profile dominated by *Firmicutes* (36.85%), followed by *Bacteroidota* (5.12%) and *Actinobacteriota* (1.20%). Within the *H. pylori*-negative gastric cancer group (Hp-GC), *Firmicutes* remained relatively prominent (37.51%), while *Proteobacteria* and *Bacteroidota* stood at 3.53% and 4.18%, respectively.

### 3.4. Taxonomic Profiling at the Genus Level and Dysbiotic Biomarkers

**Figure 5:**
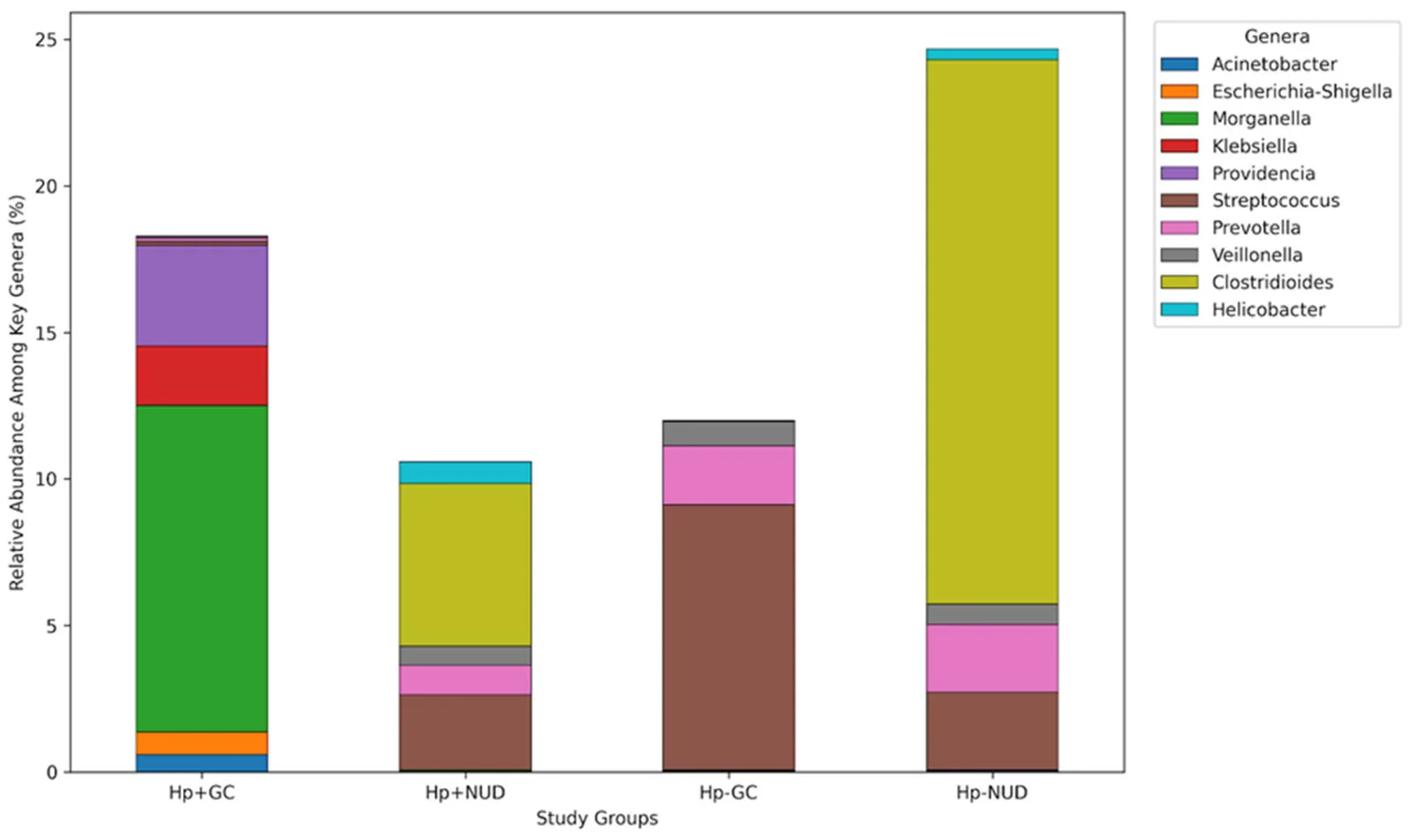
Relative abundance and distribution of dominant key bacterial genera demonstrating significant dysbiotic shifts across the study groups. Stacked vertical bars illustrate the proportional distribution of the most critically enriched or depleted bacterial pathogens and commensals. Background noise and low-frequency rare taxa (<0.1%) have been excluded to maximize visualization clarity regarding *Enterobacteriaceae* expansion (FDR-adjusted q < 0.001).

**Figure 6:**
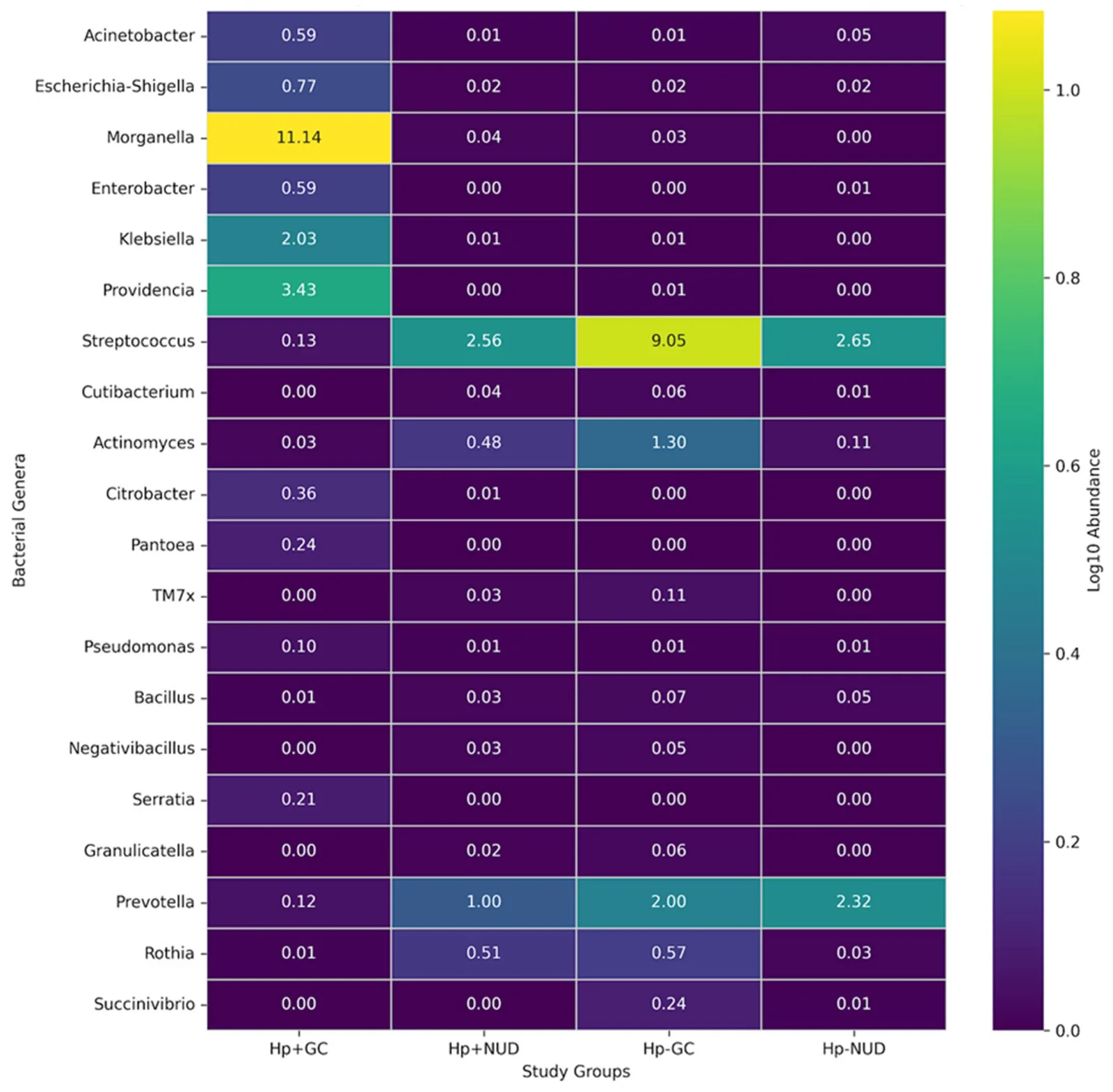
Heatmap analysis of the top 20 bacterial genera demonstrating the greatest statistical differences among the study groups. Shading intensity corresponds to log10(x + 1) transformed relative abundances across the four cohorts. The exact numerical values superimposed within each cell represent the raw, untransformed mean relative abundance percentages derived from the operational taxonomic matrices.

Genus-level analyses clearly unveiled profound dysbiotic shifts and opportunistic pathogen takeovers across the study groups (Figure 5 and Figure 6). The *H. pylori*-positive cancer group (Hp+GC) demonstrated a complete deviation from the typical gastric baseline, defined by the overwhelming proliferation of specific opportunistic Gram-negative pathogens belonging to the *Enterobacteriaceae* family. In this malignant cohort, the single most abundant genus was *Morganella*, showing a massive enrichment at a mean relative abundance of 11.14%. This was closely accompanied by other prominent opportunistic taxa, including *Providencia* (3.43%) and *Klebsiella* (2.03%). Statistical differential abundance testing confirmed that the enrichments of *Acinetobacter* (0.595%), *Escherichia-Shigella* (0.770%), *Morganella* (11.14%), *Enterobacter* (0.595%), *Klebsiella* (2.03%), and *Providencia* (3.43%) were highly significant in the Hp+GC group compared with all other cohorts (Benjamini–Hochberg FDR-adjusted q < 0.001).

Conversely, the *H. pylori*-negative cancer group (Hp-GC) was heavily dominated by *Streptococcus* (9.05% mean relative abundance, FDR-adjusted q < 0.001). In the non-ulcer dyspepsia baseline (Hp-NUD group), the anaerobic genus Clostridioides was found to be highly dominant (18.57%), representing a characteristic feature of this cohort’s non-malignant microenvironment. In the active group, Hp+NUD, an intermediate profile was observed, where commensal *Clostridioides* (5.55%) co-existed with potentially pathogenic *Streptococcus* (2.55%) and active *Helicobacter* colonization.

**Figure 7:**
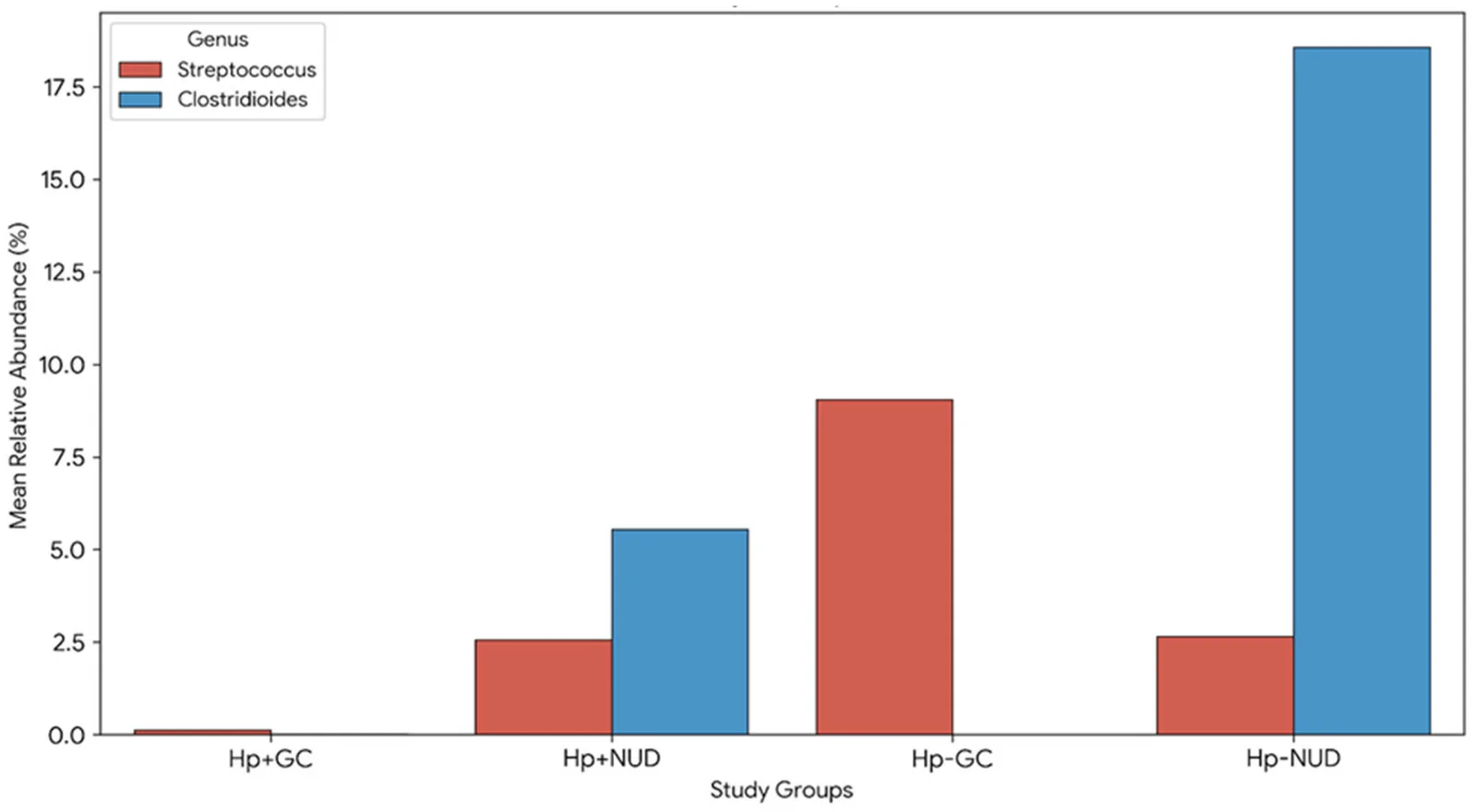
Comparison of the mean relative abundances of *Clostridioides* and *Streptococcus* genera across the study groups. Bar distributions highlight the severe, disease-specific suppression of commensal *Clostridioides* in both cancer groups and the distinct overrepresentation of oral-derived *Streptococcus* within the *H. pylori*-negative gastric cancer (Hp-GC) cohort (Benjamini–Hochberg FDR-adjusted q < 0.05).

One of the most biologically meaningful findings supporting the “hit-and-run” hypothesis was the strict distribution cascade of the *Helicobacter* genus itself. While *Helicobacter* was clearly detectable at a mean relative abundance of 0.74% in the active group, Hp+NUD, its relative abundance collapsed to negligible levels (<0.01%) within the cancerous Hp+GC tissues, despite these patients remaining clinically positive via histology and rapid urease testing. This statistically significant depletion (FDR-adjusted q = 0.045) highlights a clear dilution and replacement effect, where *Helicobacter* initiates the inflammatory Correa cascade but is outcompeted and replaced by *Morganella* and related *Enterobacteriaceae* as mucosal atrophy and hypochlorhydria neutralize the typical acidic gastric barrier (Figure 7).

## 4. Discussion

Gastric cancer (GC) is considered an inflammation-driven malignancy originating from the context of persistent *H. pylori*–associated inflammation [14]. Different *H. pylori* strains induce inflammatory responses of varying severity, whereas the altered gastric environment during carcinogenesis leads to more profound changes within the gastric microbiota [11,12]. The role of *H. pylori* in gastric carcinogenesis is well established; however, reports regarding the contribution of other members of the microbiome to disease pathogenesis remain controversial [11,12].

A pioneering study identified five major phyla—*Proteobacteria*, *Firmicutes*, *Bacteroidetes*, *Actinobacteria*, and *Fusobacteria*—in the normal gastric mucosa [8]. However, the gastric microbiota represents a dynamic ecosystem influenced by factors, including diet and medication use [14]. Decreased gastric acidity has been shown to facilitate colonization by non-*H. pylori* intestinal commensals, thereby disrupting the resident microbial balance and potentially increasing the risk of *H. pylori*-associated carcinogenesis [11].

Most studies have revealed significant differences in microbial diversity between patients with GC and healthy controls. Chen et al. [17] demonstrated that oral bacteria and lactic acid-producing species, including *Lactococcus lactis* and *Lobacillus brevis*, predominate in GC tissues. Similarly, Aviles–Jimenez et al. [18] reported a progressive reduction in bacterial diversity during the transition from gastritis to GC, whereas Coker et al. [12] described a dysbiotic profile characterized by increased oral bacteria within GC biopsies. Ferreira et al. [13] observed low *H. pylori* abundance but high intestinal commensal levels, together with reduced microbial diversity, in patients with GC. Consistent with these findings, our study revealed significantly reduced alpha diversity (Shannon index) in the Hp+GC group compared with the NUD groups (FDR-adjusted q < 0.001), thereby supporting the concept that microbial diversity declines during gastric carcinogenesis.

Gantuya et al. [19] reported that *Lactobacillus* and *Enterococcus* were the dominant genera in patients with GC and that the *Firmicutes* phylum was increased. Conversely, our findings revealed a dramatic decrease in the *Firmicutes* phylum (dropping to a mean relative abundance of 1.40%) and a marked overrepresentation of the Proteobacteria phylum (66.56%) in the Hp+GC group. Most notably, the predominance of specific opportunistic bacterial pathogens belonging to the *Enterobacteriaceae* family, showing significant enrichment of *Morganella* (11.14%), *Providencia* (3.43%), and *Klebsiella* (2.03%), represents one of the most striking results of this study (FDR-adjusted q < 0.001). These findings strongly support the hypothesis that hypochlorhydria caused by gastric atrophy facilitates stomach colonization by fecal or opportunistic pathogens.

The massive expansion of *Enterobacteriaceae*, specifically *Morganella* and *Providencia*, in the Hp+GC group suggests a multi-factorial contribution to the carcinogenic process. Opportunistic pathogens within the *Proteobacteria* phylum are known to trigger chronic inflammation by interacting with host Toll-like receptors (TLRs), leading to the continuous activation of pro-inflammatory signaling pathways [20]. Furthermore, many members of the *Enterobacteriaceae* family possess nitrate-reducing capabilities, facilitating the production of N-nitroso compounds, which are potent genotoxins capable of inducing DNA damage in gastric epithelial cells [14]. Additionally, the metabolic byproducts of these dysbiotic communities, including reactive oxygen species (ROS), may further promote genomic instability and sustain a pro-tumorigenic microenvironment [14]. These mechanistic pathways collectively suggest that the microbial shift observed in our study is not merely a consequence of environmental change but suggests that these microbial shifts may be closely associated with the progression of late-stage gastric carcinogenesis, though whether they act as active drivers or are secondary to the altered tumor microenvironment remains to be confirmed through functional studies. Moreover, the enrichment of *Enterobacteriaceae* has been consistently reported as a hallmark of gut dysbiosis in various gastrointestinal cancers. In the context of the stomach, the expansion of these facultative anaerobes is likely facilitated by the loss of the acid barrier [11]. Their role in sustaining a chronic inflammatory state through the continuous release of lipopolysaccharides (LPS) further underscores their potential involvement in promoting the malignant transformation of the gastric mucosa [20].

Comparative analyses based on *H. pylori* status and lesion presence in our study provide strong evidence supporting the “hit-and-run” hypothesis [11]. Patients in the Hp+GC group were clinically positive for *H. pylori*; however, high-throughput 16S rRNA sequencing analysis revealed that the actual relative abundance of the *Helicobacter* genus decreased to negligible levels (<0.01%). Conversely, *Helicobacter* remained sequence-detectable in the active group (Hp+NUD) at a mean relative abundance of 0.74% (FDR-adjusted q = 0.045). These findings indicate that, after initiating carcinogenesis, *H. pylori* cannot persist in the altered gastric microenvironment characterized by extensive atrophy and increased pH levels, and is subsequently outcompeted and replaced by other opportunistic pathogens, including *Morganella* [11]. The observed discrepancy between clinical positivity (via histology/RUT) and negligible sequencing reads in the Hp+GC group highlights the ‘dilution effect’ in a dysbiotic niche. This marked discrepancy between clinical diagnostic positivity and sequence-based under-representation is explained by a combination of ecological, biological, and methodological factors. Biologically, extensive glandular atrophy and intestinal metaplasia lead to the loss of specialized foveolar mucins, depriving *H. pylori* of its primary adhesion niche and resulting in patchy, low-density residual colonization as confirmed by our semi-quantitative histopathology. Methodologically, 16S rRNA amplicon sequencing generates compositional, relative abundance data rather than absolute quantitative cell counts. Our methodology also utilized 16S rRNA gene sequencing, which provides a detailed taxonomic profile but lacks direct functional validation. While we identified significant shifts in specific bacterial taxa, we cannot definitively characterize the functional pathways or metabolic byproducts—such as pro-inflammatory metabolites or genotoxins—that these bacteria might produce in the gastric niche. Consequently, as the loss of gastric acidity facilitates the massive, unchecked proliferation of potentially pathogenic *Enterobacteriaceae* (such as *Morganella* and *Providencia*), the expanded total bacterial biomass numerically dilutes the low-frequency *H. pylori* reads. Furthermore, differential PCR amplification characteristics and variations in 16S rRNA operon copy numbers across divergent taxa may further suppress the apparent relative yield of low-abundance organisms within a complex polymicrobial mixture. While *H. pylori* remains present at a cellular level, its relative abundance in the total microbial community is drastically reduced as opportunistic pathogens proliferate in the hypochlorhydric cancer environment. Consistent with our results, Liu et al. [21] reported significantly reduced *H. pylori* abundance in tumor tissues, indicating that microbial diversity may differ even between tumor and adjacent nontumor tissues within the same patient.

With progressive gastric atrophy, *H. pylori* colonization decreases and gastric acidity declines, thereby enabling the survival of nitrosating bacteria or opportunistic pathogens, including *Morganella*, as observed in our study [11]. Ammonia and bicarbonate produced from urea metabolism by *H. pylori* may serve as substrates for other bacteria, thereby facilitating the formation of new microbial niches [11]. Similarly, Karnes et al. [22] revealed that *H. pylori* was present in active gastritis but absent in advanced GC despite serological positivity.

Another hypothesis states that members of the Nitrospirae phylum may contribute to gastric carcinogenesis through nitrite production [23]. Alternatively, lactate produced by *Lactococcus* and *Lactobacillus* may serve as an energy source that promotes tumor growth and angiogenesis [24]. The interaction between bacteria and the host immune system, mediated through TLRs and NOD-like receptors, may further contribute to carcinogenesis by inducing chronic inflammation [20]. While our taxonomic findings provide a robust framework for understanding the structural shifts in the GC microbiota, the precise functional roles of dominant bacterial taxa like *Morganella* remain speculative. Future studies employing shotgun metagenomics and metabolomics are critically needed to validate the active functional pathways and metabolic byproducts (e.g., genotoxins or pro-inflammatory metabolites) of these dysbiotic communities, thereby bridging the gap between taxonomic presence and biological activity.

**Figure 8:**
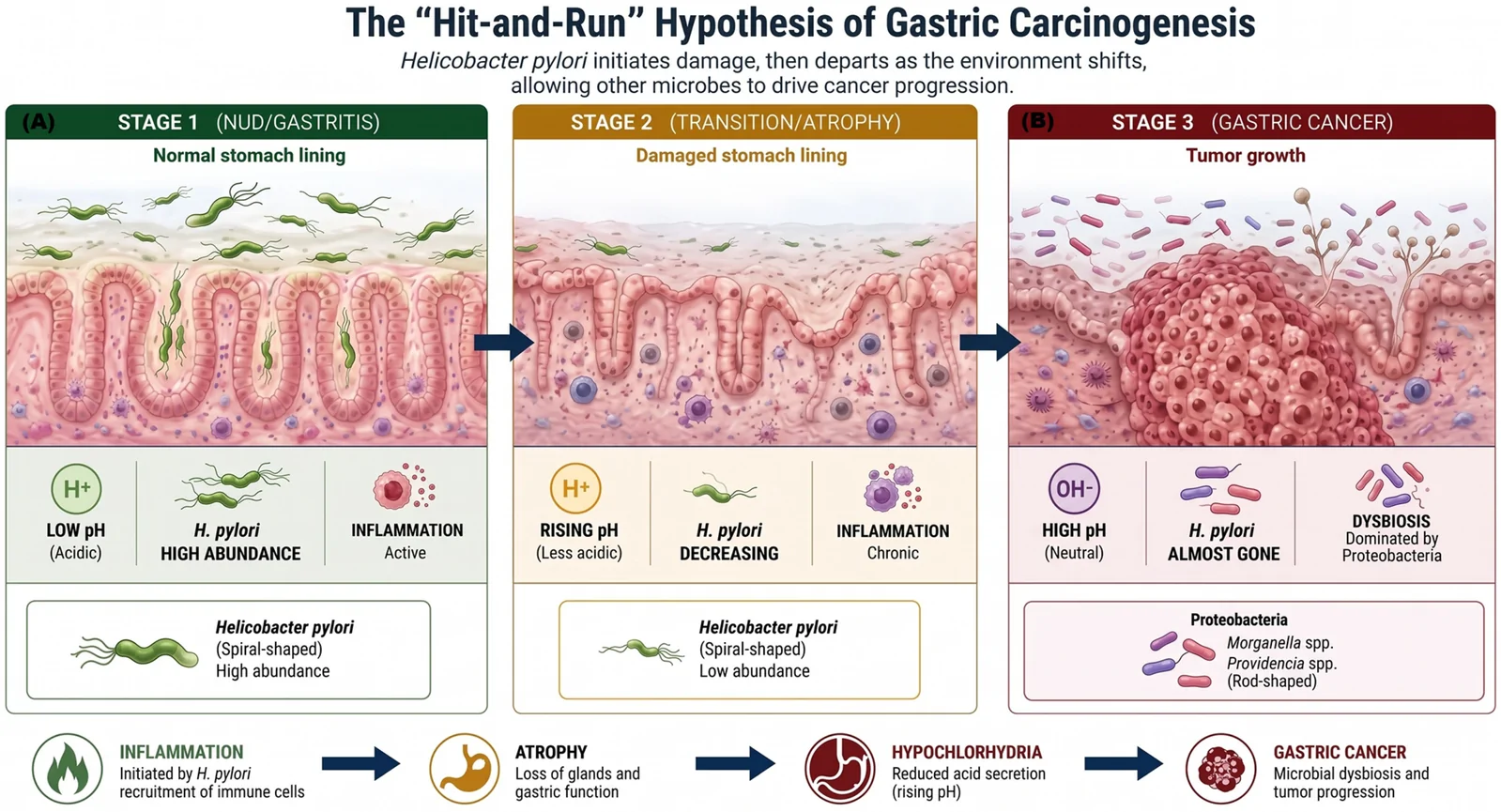
Schematic representation of the gastric microbial “hit-and-run” mechanism during the sequential progression to gastric cancer. The conceptual model illustrates the clinical transition from (**A**) a non-ulcer dyspepsia (NUD) state, characterized by physiological gastric acidity and robust *H. pylori* colonization, to (**B**) a gastric cancer (GC) malignant state. Chronic, pathogen-driven inflammation precipitates widespread mucosal atrophy and a subsequent elevation in intraluminal pH (hypochlorhydria). This structural breakdown of the acid barrier forces the depletion of the primary initiator, *H. pylori* (the “run” phase), and enables the aggressive colonization and overgrowth of opportunistic, nitrate-reducing bacterial pathogens belonging to the *Enterobacteriaceae* family (e.g., *Morganella*, *Providencia*, and *Klebsiella*), sustaining a chronic pro-tumorigenic microenvironment.

Figure 8 illustrates the proposed schematic model of the gastric ‘hit-and-run’ mechanism. As the gastric microenvironment evolves from chronic inflammation to malignancy, the loss of parietal cells and subsequent rise in pH lead to the depletion of *H. pylori* and the establishment of a dysbiotic community dominated by specific potentially pathogenic *Enterobacteriaceae* members.

In our gastric cancer cohort, 37 of 62 patients (59.7%) tested positive for *H. pylori* by Giemsa staining and/or rapid urease testing, whereas 25 (40.3%) tested negative. Published positivity estimates vary across gastric cancer cohorts. Boixeda et al. [25] reported *H. pylori* infection in 41% of 54 patients with gastric adenocarcinoma, while a meta-analysis of 55 studies comprising 5324 patients with non-cardia gastric cancer in China reported a pooled prevalence of 66.5% (95% CI, 62–71%) [26]. Our estimate lies between these figures, but the cohorts are not directly comparable because of differences in tumor location, study population, sampling, and infection assessment. In particular, a negative tissue-based test at cancer diagnosis does not establish that a patient has never been infected with *H. pylori*. 

This study has certain limitations, including a relatively limited sample size and the cross-sectional nature of biopsy sampling [15]. Specifically, the cross-sectional design restricts our ability to observe the temporal evolution of the gastric microbiota within individual patients over time. Considering the dynamic nature of the gastric microbiota and its susceptibility to dietary and pharmacological influences, future longitudinal data remain essential to provide definitive functional evidence and confirm the causal nature of the transitions observed during the progression from NUD to GC [14]. The distribution of gastric cancer subtypes differs substantially between the Hp+GC and Hp−GC groups, which also may be considered as a limitation. In the Hp+GC cohort, 75.68% of tumors were intestinal type and 24.32% diffuse type, whereas the Hp−GC cohort contained 20% intestinal-type and 80% diffuse-type tumors. This difference may be relevant because histological subtype could potentially influence the gastric microenvironment and microbial composition. We used NUD patients as a non-malignant control group, since obtaining biopsies from healthy patients is ethically objectionable. DNA extracts from the antrum and corpus were pooled to obtain a single patient-level microbial profile and to avoid pseudoreplication. However, this approach prevented the assessment of site-specific differences in microbial composition, which may be relevant because *H. pylori* colonization and gastric atrophy can vary between anatomical regions. Nevertheless, other factors, including longer-term medication history, diet, smoking, alcohol consumption, comorbidities, and other gastrointestinal conditions may also influence microbial composition. We had no data for these factors, which we consider to be a limitation.

However, our study possesses several important strengths. First, patients were stratified not only by cancer status but also by *H. pylori* infection into four homogeneous cohorts (Hp+GC, Hp−GC, Hp+NUD, and Hp−NUD), thereby enabling precise discrimination of microbiota alterations. Second, despite the cohort boundaries, the strong statistical significance (FDR-adjusted q < 0.001) and large effect sizes observed indicate that the identified “hit-and-run” pattern—characterized by *Morganella* dominance—is a noteworthy and robust biological signal within the gastric niche. Finally, the rigorous implementation of negative controls processed alongside each clinical batch markedly reduces the likelihood of exogenous kit- or environment-derived contamination artifacts, supporting the biological authenticity of our findings.

## 5. Conclusions

Our findings reveal that gastric carcinogenesis is a dynamic process driven by *H. pylori*-induced environmental alterations rather than by the single pathogen alone. The drastic reduction in the *Helicobacter* genus to near-absent levels and the concurrent profound dominance of specific potentially pathogenic *Proteobacteria* (most notably *Morganella*) within the *H. pylori*-positive gastric cancer group are consistent with the “hit-and-run” theory of gastric carsinogenesis. This gastric microhabitat alteration, characterized by extensive mucosal atrophy and a neutralized intraluminal pH (hypochlorhydria), leads to the structural loss of *H. pylori* and facilitates aggressive colonization by non-acidophilic opportunistic bacterial pathogens. Consequently, the severe dysbiotic shift defined by *Bacteroidota* depletion and non-*Helicobacter* Proteobacterial proliferation represents a prominent biological signature that is intimately linked to the later stages of gastric tumor progression. 

Future clinical and therapeutic approaches may consider targeting this highly dysbiotic bacterial microbial community as part of comprehensive gastric management, alongside standard *H. pylori* eradication protocols. This approach should be seen as a potential future therapeutic avenue rather than as an implication directly established by the current study. However, given the cross-sectional nature of this study, these associations should be interpreted cautiously, and further longitudinal and functional research remains essential to definitively determine whether these specific bacterial shifts play an active causal driving role in driving carcinogenesis or act as secondary successions resulting from the altered tumor microenvironment.

## Figures and Tables

**Table 1:** Demographic data and laboratory findings (endoscopic, histopathological, and microbiological findings) of the study and NUD groups.

Demographic Data and Laboratory Findings	Study Groups (SG)		NUD Groups	
	Hp (+) Gastric Cancer (GC)	Hp (−) Gastric Cancer (GC)	Hp (+) Non-Ulcer Dyspepsia [NUD Hp (+)]	Hp (−)] Non-Ulcer Dyspepsia [NUD Hp (−)]
Subject (*n*)	37	25	17	17
Mean Age (min–max)	68.7 ± 9253 (43–79)	67.96 ± 8.73 (55–79)	67.32 ± 10.65 (55–78)	66.93 ± 8.83 (59–75)
Gender Male (%) Female (%)	23 (62.17%) 14 (37.83%)	14 (56.0%) 11 (44.0%)	11 (64.71%) 6 (35.29%)	11 (64.71%) 6 (35.29%)
Site of Involvement *n* (%)	Intestinal type 28/37 (75.68%)	Intestinal type 5/25 (20.00%)		
	Diffuse type 9/37 (24.32%)	Diffuse type 20/25 (80.00%)		

## Data Availability

The raw sequencing data (FASTQ files) supporting the findings of this study are maintained on secure institutional servers due to ethical and legal restrictions related to the protection of clinical data (Istanbul University, Cerrahpasa Clinical Trials Ethics Committee, No: A-57, date: 2 April 2019). However, anonymized datasets, including taxonomic feature tables and metadata, are available from the corresponding author upon reasonable request.
